# Impact of Position in Family on Suicide: A Case-Control Psychological Autopsy Study among Chinese Rural Youths

**DOI:** 10.1155/2023/1797697

**Published:** 2023-09-16

**Authors:** Qin Zhou, Ning Li, Jie Zhang, Libo Fan

**Affiliations:** ^1^School of Public Administration and Policy, Renmin University of China, China; ^2^Institute of Population Research, Peking University, Beijing, China; ^3^Central University of Finance and Economics, Beijing, China; ^4^State University of New York College at Buffalo, USA; ^5^Business School, University of International Business and Economics, Beijing, China

## Abstract

**Introduction:**

Suicide remains an important public health issue in China. Existing literature on the relationship between individual-familial variables and suicide risk mainly focused on family socioeconomic status, and few studies analyzed the effect of position in family on suicide. In this study, we aimed to explore the association between position in family and suicide among Chinese rural youths.

**Methods:**

We conducted a case-control psychological autopsy study. The data collection yielded 392 suicide cases aged 15-34 years and 416 community living controls within the same age range. Personal position in family was assessed by the question “How do you evaluate his/her position in the family?” and categorized as high, general, and low to reflect the relative position in family. Logistic regression models were conducted to explore the association between position in family and suicide risk.

**Results:**

The results showed that compared with females (males) having a high position in family, females (males) with a low position in family were 7.1 (9.1) times more likely to commit suicide (*p* < 0.01). Mental disorders, social support, and coping strain were potentially important mediating factors linking position in family to suicide, with certain heterogeneity among males and females. Low coping strain played the most important role in underlying the association between a low position in family and suicide for both females and males, accounting for 55% (28%) of the contribution to the total effect for females (males). Subgroup analysis revealed that low position in family had more significant impacts on suicide risk among married youths and those with low education levels.

**Conclusion:**

The effect of position in family on suicide should receive greater consideration when predicting suicide in rural China. Possible mechanisms underlying the effect of position in family on suicide include mental status, social support, and coping strain.

## 1. Introduction

Suicide remains an important public health issue in China, although the suicide rate has decreased substantially over the past decades [1]. In 2017, the estimated mean suicide rate was 7.2 per 100,000 persons in China, which has declined by 65% compared to the rate of 20.9 per 100,000 persons in 1990 [2]. However, there was still an upward trend in 15–34 rural males in suicide rates from 2005 to 2019 [3].

Studies conducted in China identified some unique risk factors for suicide, such as being female [4], rural residence [5], single status [5], religious belief [6], mental disorders [6], high chronic stress [7], low social support [8], high impulsivity [9], severe psychological strain [10], conflicts with family members [5], and previous suicidal behavior [11]. A growing body of studies used the strain theory of suicide to explain suicide in China and showed evidence that psychological strains were significantly associated with suicide [2, 12, 13].

For suicide risk factors at the individual-familial level, most studies explored the impact of socioeconomic status (SES) on suicide, and few studies analyzed the impact of position in family on suicide. For example, Kim et al. studied the relation between socioeconomic inequalities and suicidal ideation, parasuicide, and completed suicide in South Korea [14]. They found that both absolute and relative inequalities in socioeconomic position were highly correlated with suicidal ideation and mortality. Moreover, Iemmi et al. conducted a systematic review to understand the association between suicidal ideations, behaviors, and economic poverty in low-income and middle-income countries [15]. They found that poor economic status, diminished wealth, and unemployment were associated with suicidal ideations and behaviors. There are few studies on the relationship between socioeconomic status and suicide risk among the Chinese population. For example, low educational level, unemployment, and low income were suggested to be risk factors behind suicide [12, 16]. Zhang and Tao investigated the relationship between family socioeconomic status and the psychopathology of Chinese college students and found that perceived family socioeconomic status was positively correlated with suicide ideation among college students [17].

In this study, we aimed to provide new evidence of the impact of personal position in family on suicide risk among Chinese rural youth. There is politics in the family, which is called family politics. Politics is not only about who is more powerful in the family but also about whether family members can be harmonious. In the power structure of this family, some members are more powerful in the family, while some are in a disadvantageous position. Mental disorders and suicide are often the result of games of power in family politics [18]. In the power structure of this family, family members have different contributions to the family, and thus their positions within the family might be different. There are many factors that could affect the family position which might be related to perceived honor, finances, gender, family roles, and so on. Different individuals and families may value family position from different aspects, and the data in this study reflects the subjective general estimation from the respondents on family position. Recent psychological and sociological researches have shown that subjective experience has direct impacts on personal behaviors, such as personal health and health behavior [19].

This study focused on the general estimation of family position from family members, and the factors affecting the estimation of family position warrant future researches. We also explored potential mediating mechanisms for the relationship between position in family and suicide risk from the perspectives of mental health, social support, and coping strains, which were neglected in the existing literature.

We used the psychological autopsy (PA) method to compare the position in family between young adults who committed suicide and living controls from the same village. The PA method, known as “a procedure for the reconstruction of suicidal death through interviews with survivors” [20, 21], has been widely used in Western countries for suicide studies. This method is shown to be an equally applicable method for the study of completed suicides in China [16] and has been suggested to be the optimal research method given the environment of Chinese rural suicides [22].

## 2. Methods

### 2.1. Study Population and Design

The data for this study was derived from a case-control psychological autopsy study. The survey was designed to explore potential risk factors for suicide among Chinese rural young adults, such as mental disorders, impulsivity, and strain. The target population of the study was rural young adults aged 15-34 years old who died by suicide in comparison with community-living controls from the same village. In this study, we focused on the association between family position and suicide risk.

### 2.2. Sampling

The survey was carried out in three provinces in China, including Liaoning, Hunan, and Shandong. A total of 16 counties were randomly selected in the 3 provinces, with 6 in Liaoning, 5 in Hunan, and 5 in Shandong. Within each county, all village doctors were trained on study procedures and were required to report suicide deaths to the local Centers for Disease Control and Prevention (CDCs) by telephone or fax within 24 hours after the suicide occurred. If suicidal deaths were not recognized by any health agency, village treasurers (village chair), who collected fees for each burial or cremation and were aware of all deaths in the village, were required to notify the county CDC. To ensure that no cases of suicide were missed, we conducted investigations with the village board and villagers whenever necessary. Subsequently, all the suicide information gathered at the county CDCs was transferred monthly to the provincial CDC. Finally, we collected 393 suicide cases among 15-34-year-olds from October 2005 to June 2008, with 178 female and 214 male subjects.

We randomly selected living comparison individuals within the same age range (i.e., 15-34 years) and the same county, based on the 2005 census database of the 16 countries. Finally, 416 comparison subjects were collected, with 214 females and 202 males.

### 2.3. Information Sources

For each suicide case and each control, we carefully selected two informants to interview who were 18 years or older and most familiar with the subject's life and circumstances. The first informant was a parent, a spouse, or an important family member. The second informant was a friend, coworker, or neighbor. The comparison individuals were also interviewed.

### 2.4. Interviewing Procedures

Informants were first approached by the local health agency or the village administration and were notified of the upcoming interview. Upon their agreement through written informed consent, the interview was arranged 2-6 months after the suicide. Informants had the opportunity to decline participation, and none of them declined participation. The control and informants were interviewed face to face by the trained interviewer in a private place in a hospital/clinic or their home. The average duration time for each interview was 2.5 hours.

### 2.5. Measures

The main outcome variable in this study was the dummy variable of suicide risk. The key variable of interest was the personal position in family, which was assessed by the question “How do you evaluate his/her position in the family?” Respondents were asked to tick one out of the five proposed answers: “highest/higher/general/lower/lowest.” We combined the first two categories into “high,” and the last two into “low.”

Personal economic status in a family is measured by personal annual income, whether an individual has income or not, and whether individual income is higher than the family average level or not. We estimate the role of personal economic status in the family on suicide risk to identify whether the impact of personal position in family on suicide stems from personal economic status in family.

Control variables in the analysis included gender, age, educational level, marital status, work status, and family economic status. Age was set as a dummy variable: “<25” and “≥25.” Educational level was categorized into three groups: “elementary school and below,” “primary school,” and “high school and above.” Marital status was categorized into two groups: “currently not married” and “currently married.” Work status was dichotomized as “employed” and “not employed.” Family economic status was measured by family annual per capita income with the Chinese renminbi (yuan), which was divided into three levels: “<10,000 yuan,” “10,000-19,999 yuan,” and “≥20,000 yuan” (one USD was equivalent to about 7.00 RMB at the time of the survey).

Furthermore, this study tried to explore potential mediating mechanisms for the relationship between position in family and suicide risk from the perspectives of mental health, social support, and coping strains. First, mental disorders were defined as 1 if the case/control had at least one category of mental diseases diagnosed by psychiatrists and 0 if otherwise. We used the Chinese version of the Structured Clinical Interview for the DSM-III-R (SCID) to measure axis I diagnoses for cases/controls [23, 24]. Diagnoses were made by the psychiatrists on each interview team in consensus meetings at which all responses from each informant were presented by the interviewers. Five categories of mental disorders were diagnosed, including mood disorders, schizophrenia and other psychotic disorders, anxiety disorders, substance use disorders, and other axis I disorders (stress-related disorders, eating disorders, somatoform disorders, pathological gambling, and adjustment disorders).

Second, social support was assessed with the perceived social support subscale in the Duke Social Support Index (DSSI) [25], which reflects the participants' perception of his or her social support from family members and friends (e.g., “Do you feel useful to family and friends?”). The level of social support was divided into three groups based on the 33.3rd and 66.6th percentiles (scores = 15 and 19, respectively) among the whole sample. The Chinese version of the DSSI has been validated in earlier studies [26, 27].

Third, the coping strain was measured by the Coping Responses Inventory (CRI), which was developed by Moos [28]. When facing a life crisis, people would experience coping strain if they were not able to cope with it [10]. Subjects were assumed to suffer coping strain if they had low coping skills. The CRI asked respondents to evaluate the target's frequency (0 = never, 1 = occasionally, 2 = sometimes, and 3 = often) of engaging in 48 skill activities. Out of the 48 items, 24 measured positive skill activities which were protective factors of suicide (e.g., “Know what has to be done and try hard to make things work”) and 24 measured negative ones reflecting risk factors (e.g., “Accept it; nothing can be done”). The responses to questions on negative skill activities were assigned to an integer value of 0 to 3, and the responses to the questions on positive skill activities were recoded as 3 to 0. Finally, we obtained total scores of coping strain by adding up the overall score of the 48 items, with higher scores indicating higher coping strain. The approach coping subscale has been tested in the rural Chinese population with good validity and reliability [10, 29].

PA was a systemic method to study risk factors preceding suicide and was commonly used in Western countries [30, 31]. Information on demographics, family and society circumstances, and personal psychological status was collected from proxy respondents which made the information relatively objective [30, 31]. In PA studies, including the current one, many information was collected via the informants of participants. Therefore, the validity of using proxy data and the reliability and validity of the methodology and instruments were tested before the study [30–32]. The results indicated that PA was a valid method to study suicide in China [30–32], and the information provided by the informants might not be perfect but was reliable for PA study in China [30–32].

### 2.6. Statistical Analyses

STATA (version 15.0) was used for data analysis. *T*-test and chi-square tests were used to compare differences in continuous and categorical variables between cases and controls, respectively. For study outcome part 1, we employed multiple logistic regression models to estimate the coefficients of position in family in relation to suicide, adjusting for individual and family characteristic factors.

For study outcome part 2, we tried to distinguish the role of personal economic status and noneconomic status in the family on suicide risk. For the first step, we added separate variables of personal annual income and personal relative income in the family instead of personal status in the family to estimate the effect of personal economic status in the family on suicide risk.

Since personal status includes the individual's economic and noneconomic status, the coefficient of personal status in the family represents the role of personal noneconomic status in the family if personal economic status in the family is controlled. For the second step, we added both measures of personal status in the family and the personal economic status in the family within the model to estimate the coefficient of personal status in the family after adjusting for personal economic status in the family. If the coefficient of personal status in the family remains significant, it provides evidence that personal noneconomic status in the family plays a significant role in suicide risk. Hence, in the second step, we added variables of individual income, whether one has individual income or not, and whether individual income is higher than the family average level or not into the model gradually from the first step to estimate the role of individual economic position on suicide risk. This result would help to interpret whether the effect of position in family on suicide risk is related to personal economic status.

For study outcome part 3, we performed sensitivity analyses by examining heterogeneity among subgroups by marital status and educational level. For study outcome part 4, we employed the strategy of identifying mediating mechanisms using the stepwise regression method. We added covariates gradually into the model from the first step and then examined the association among those covariates and suicide risk, as well as how the coefficients of position in family would change. The final model included all indicators simultaneously. The added covariates included mental health, social support, and coping strain. For the strategy of identifying a mediating mechanism, if the covariate is added into the model and the coefficients of position in family become smaller or less significant, then the covariate is proved to be an important mediator in the pathways of position in family affecting suicide risk. This strategy of identifying mediating mechanisms has been widely used in the literature [33–35]. In addition, we employ the Karlson-Holm-Breen (KHB) method which was developed by Karlson et al. [36] to further explore the contributions of mediators to the association between low position in family and suicide risk. We also disentangle the contributions of mediators to identify which of the mediators contribute most to the confounding.

## 3. Results

### 3.1. General Information

The data collection yielded 392 suicide cases and 416 community living controls in rural China. All the subjects were aged between 15 and 34 years at the time of death or interview. As shown in Table 1, suicide cases were more likely to have a low position in family. For example, 11.24% and 16.82% of female and male cases had lower positions in family, while the corresponding proportions for living females and males were only 1.87% and 1.49%, respectively.

Suicides were more likely to occur among the unmarried. There was no significant difference in working status between suicide cases and controls. Both suicide females and males had lower educational levels and were more likely to be from families with lower annual income than controls. There was no significant difference in personal annual income between female cases and female controls, while male cases had significantly lower annual income than their controls. Compared with living females, more female cases had a lower annual income than the family average annual income (PI < FI), while there was no significant difference between male cases and their controls. Compared with living controls, more cases had mental disorders, lower social support, and lower coping skills.

### 3.2. Multivariate Analysis

#### 3.2.1. Impact of Position in Family on Suicide Risk

We performed logistic regressions to estimate the association between position in family and suicide for females and males separately and reported ORs in Table 2. The table showed suicide risk estimates for demographic factors, family income, and position in family, with the first two columns for females and the last two columns for males.

As Table 2 illustrates, males aged 25 years and above had a significantly higher risk of suicide than males younger than 25 years old (see column 4), while it was not significant among females (see column 2). Lower educational levels and unmarried status were strong predictors of suicide, and the effects were stronger among males than among females. Family economic status was highly associated with suicide. Females and males from low-income families (RMB < 10000) were more likely to commit suicide than individuals from high-income families (RMB ≥ 20000).

Column 2 and column 4 in Table 2 showed that the ORs of low position in family were 7.1 (*p* < 0.01) for females and 9.1 (*p* < 0.01) for males. In other words, compared with females (males) having high positions in family, females (males) with low positions in family were 7.1 (9.1) times more likely to commit suicide.

#### 3.2.2. Role of Personal Economic Status on Suicide Risk

Table 3 showed that the coefficients of personal economic status variables (including individual absolute income, having individual income or not, and income higher than family average level or not) were not significant at the 5% level both for females (see columns 2-4) and males (see columns 6-8), and coefficients of position in family remained the same as those in Table 2 (columns 2-4 for females and columns 6-8 for males). It indicated that personal income was not directly associated with suicide risk after controlling for family income per capita income and other control variables, indicating that the impact of personal position in family on suicide did not stem from personal economic status.

#### 3.2.3. Subgroup Analysis

Probing for heterogeneity (Table 4), we found that position in family had a more significant impact on suicide risk among the married population (OR = 32.59, *p* < 0.01) than among the unmarried population (OR = 3.50, *p* < 0.01). For education heterogeneity, we found that the impact of low position in family on suicide risk was strongest among people with a primary school level of below (OR = 17.33, *p* < 0.01), followed by people with a middle high school level (OR = 7.03, *p* < 0.01), and people with a senior high school level and above (OR = 4.78, *p* < 0.01).

#### 3.2.4. Mediating Factors

Furthermore, we explored the association between mental health, social support, coping strain, and suicide risk and examined how the coefficients of position in family would change when these variables were added to the model. In Table 5, we added the above variables gradually to identify the potential mediators for females in columns 1-4 and males in columns 5-8. (Take the female model as an example, in column 1 of Table 4, only control variables and the explanation variable (position) were included in the model. In columns 2-4, new covariate variables (variables of potential underlying mechanism) were added into the model gradually, including mental disorder, social support, and coping strain. In column 4, all the covariates were included in the model.)

We added the variable of mental disorders in column 2 for females and column 6 for males and found that females and males with mental disorders were 13.36 times and 32.51 times more likely to commit suicide in comparison to living subjects, respectively (see column 2 for females and column 6 for males). The OR magnitude and significance of low position in family declined after the variable of mental disorders was added in the female model (OR declined from 7.09⁣^∗∗^ to 3.81⁣^∗^), while the corresponding coefficient was barely affected among males (see columns 5 and 6). It indicated that a low position in family might influence suicide risk by affecting the mental health of females.

We further added social support variables in column 3 for females and column 7 for males in Table 5. Social support was significantly associated with suicide risk for both females and males. For females, the coefficient of low position in family declined substantially after controlling for social support variables and was no longer significant (OR declined from 3.81⁣^∗^ to 1.19). For males, there was also a sharp decline in the coefficient of low position in family (OR declined from 9.60⁣^∗∗^ to 4.89⁣^∗^). The above results indicated that social support might play an important role in mediating the effect of a low position in family on suicide for both females and males.

Finally, we added the coping strain variable in column 4 for females and column 8 for males. Females and males having high coping strain (low coping skills) were 29 times and 18 times more likely to commit suicide than living controls, respectively. The coefficient of low position in family for males declined to a certain extent after controlling for coping strain (OR declined from 4.89⁣^∗^ to 3.71⁣^∗^). It indicated that coping strain could be a partial mediator in the association between low position in family and the risk of suicide for males.

Based on the stepwise regression method to study the mechanism effect, we further employ the KHB method [37] to explore the contributions of mediators to the association between low position in family and suicide risk. Tables 6 and 7 reported the average partial effects of a low position in family on suicide before and after controlling for potential mediating factors. On average, the probability of suicide was 13.7 times higher for females with a low position in family compared to those with a high position in family. After controlling for mental health, social support, and coping strain, the difference in suicide probability was no longer significant among individuals with low and high positions in family. Similarly, after controlling for the above three potential mechanisms, the probability of suicide reduced from 34 times to 4 times for males with low positions in family, and the corresponding significance was reduced to a 5% significance level. The above results indicated that mental health, social support, and coping strain are the main mediators underlying the association between suicide risk and low position in family for both females and males.

Further, we explore which mediators contribute most to the confounding in Table 7. Column 1 for females and column 2 for males showed the contribution of each mediator to the indirect effect (the overall confounding by all mediators together). The results indicated that low coping strain played the most important role underlying the association between low position in family and suicide risk for both females and males, and mental disorders problem contributed to the second, and the third one is low social support.

## 4. Discussion

This study provided new evidence that position in family was significantly associated with elevated suicide risk. Females (males) with low positions in family were 7.1 (9.1) times more likely to commit suicide compared to females (males) with high positions in family. It indicates that position in family is significant in predicting suicide among Chinese young adults. Our results were consistent with the findings of Fei [18], which indicated that suicide was often a result of games of power in family politics. On the one hand, when the power balance is broken in the family and family harmony is unsustainable, suicide might happen. On the other hand, if one family member had an obviously disadvantageous position with less moral capital and economic capital, in other words, he/she owned low power in the family, thus he/she would be confronted with a higher risk of extreme behaviors including suicide. Our study provided evidence that subjective experience of family position has direct impacts on suicide behaviors, which is consistent with Simandan's [19] findings. Our results also showed gender differences, with males more likely to be affected by family position which could be due to the fact that males value “face” more than females do in China [38].

We have to mention that low position in family and suicide may affect each other. Personal position in the family of people who died of suicide might be rated low due to stigmatization, a phenomenon that was common among patients with mental disorders [39, 40] and people who died of suicide [41, 42]. For example, patients with mental disorders might perceive stigma from other family members [40], and family members might also feel ashamed of suicide behaviors in China [43]. The stigma problem is therefore a big challenge for psychological autopsy studies on suicide.

We also provide new evidence that personal economic status in a family is not significantly related to suicide risk after controlling for family income, personal position in the family, and other factors. One possible reason might be the impact of household division of labor. The Chinese culture has a gender division of household labor in the family, especially in Chinese rural families. Normatively, the males head the family, and they are supposed to take charge of external matters and be responsible for the orderly management of the family, while the females have to shoulder more domestic work, such as cooking, laundry, and taking care of family members [44–46]. Family members who fail in their “expected tasks” (such as those who are assigned to stay home with the kids), hence, would encounter a lower position in family regardless of their income. Our findings indicate that in the Chinese traditional context, personal noneconomic status in the family might be more important in predicting suicide risk than economic status. However, we could not rule out the possibility that the general average household/personal income in the locations where the study was carried out has very little variability itself. Hence, our results on the effect of personal economic status in family on suicide risk should be interpreted with caution, and more robust evidence is needed in future research.

Subgroup analysis revealed that low position in family had a more significant impact among the married population. A possible explanation might be that the transition from unmarried to married brought more complicated family relationships, and people with low positions in family might undergo negative effects from the other members of the family. If appropriate coping skills and social support are absent, these young married people might become vulnerable and be at high risk of suicide.

Results of subgroup analysis also indicated that the impact of low position on suicide risk was stronger among people with low education levels. This might be explained by their low income level, tough life, lack of social support, and limited coping skills. The government and social organizations should pay more attention to this disadvantaged population and provide them with psychological counselling and support.

Furthermore, we identified the potential mechanism underlying the association between position in family and suicide risk, which was neglected in the existing literature. Our results indicate that mental disorders, low social support, and low coping skills might be the potential mediating factors that link low position in family to suicide. In the power structure of this family, when family conflicts happen, both female and male young adults with low family positions easily plunge into complex and confusing personal dilemmas. This could cause strong mental stress and even mental disorders. If they have a low level of social support and inadequate coping skills, they might conduct extreme behaviors. For suicide intervention, our findings suggest that family education on how to maintain family harmony, to deal with family conflicts, and to improve coping skills might be useful. For families with severe family conflicts, local community officers should pay more attention and provide help in mediating and counselling.

The findings in this study have significant implications for future suicide prevention/intervention and future research. First, for families with severe conflicts among family members, the community neighborhood committee should pay more attention and provide prompt help and psychological counselling. This could help dilute family conflicts and avoid extreme risky behaviors. Second, interventions for family members with low family positions should be considered. For example, we find that young married people with low family positions are at higher risk of suicide behavior, which indicates that education programs and counselling on marriage and family are quite important. The government and social organizations could provide training classes for young married couples on how to get along with their spouse's family members, how to adapt to the new family, how to deal with family conflicts, etc. In addition, families with lower economic status tend to have more life difficulties which might lead to family tensions, and people with lower income levels usually have limited education attainment and coping skills to deal with family conflicts. Therefore, more welfare, social security, and social support should be provided to these disadvantaged people. Third, for precise and tailored suicide prevention, identifying factors associated with low family position is important, as this might give further clues to the mediators. There are many factors that might be related to one's family position. For example, some family members might be the main source of income for the family and thus earn a high position in family [47]; some family members might have high moral capital and reputation in the family which could help to maintain and improve family harmony and thus might have a high position in family [48]; in some families with son preference, women might have a low family position if they gave birth to daughters and had no son [49, 50]. The reasons for the low family position might be complex which warrants further investigations.

There were several limitations in this study. First, the nature of the case-control design could result in recall bias due to the retrospective assessments by the participants. Besides, the participants might be emotional and hard to be interviewed due to bereavement shortly after the suicide death. Therefore, we conducted the interview 2-6 months after the suicide death, when the impacts of bereavement and recall bias were relatively small. The problem of recall bias is also a major challenge for other psychological autopsy studies of suicide [51]. Second, we explored three potential mediators underlying the association between position in family and suicide risk. Despite the evidence, the coefficients should not necessarily be interpreted as causal effects because mediating factors and an individual's position in family are correlated. Thus, a causal inference is necessary for further study, and more work needs to be done with respect to the gender difference in mediating mechanisms. Third, the data used in this study was collected nearly ten years ago. China was one of the few countries where suicide rates were higher among females than among males, and suicide rates have declined with an increased male-to-female ratio in the past decades [3]. However, the gender ratio of 1.56 is still smaller than that in Western countries where the ratio is nearly 3 to 4, which means female suicide rates are yet relatively high in China [2]. Therefore, the findings in this study are still meaningful for suicide prevention among Chinese females. Future studies using more recent data to further examine the association between family position and suicide are needed.

To the best of our knowledge, this is the first study exploring the association between personal position in family and suicide risk among the Chinese population. The results demonstrate a significant impact of personal position in family on suicide risk among Chinese rural young adults. People with lower positions in family suffer a higher suicide risk. We also provide evidence that the possible mechanisms underlying the effect of personal position in family on suicide risk include mental status, social support, and coping strain. The findings have important implications for suicide prevention in China and other developing countries; tailored intervention within the family should be considered, and more attention and help for family members with low family positions from the community and society are warranted.

## Figures and Tables

**Table 1:** Descriptive statistics among suicide cases and controls.

Variables	Female (*n* = 392)			Male (*n* = 416)		
	Suicides (*n* = 178)	Controls (*n* = 214)	*p* value	Suicides (*n* = 214)	Controls (*n* = 202)	*p* value
Age (years)^a^	26.80 (6.06)	25.91 (6.19)	0.15	26.92 (6.54)	25.47 (6.13)	0.02
Marital status			0.15			<0.01
Currently married	116 (65.17%)	154 (71.96%)		82 (38.32%)	112 (55.45%)	
Not currently married	62(34.83%)	60 (28.04%)		132 (61.68%)	90 (44.55%)	
Educational level			<0.01			<0.01
Primary school and below	75 (42.13%)	38 (17.76%)		95 (44.39%)	25 (12.38%)	
Middle school	84 (47.19%)	125 (58.41%)		100 (46.73%)	127 (62.87%)	
High school and above	19 (10.67%)	51 (23.83%)		19 (8.88%)	50 (24.75%)	
Working status			0.45			0.34
Unemployed	62 (34.83%)	60 (28.04%)		127 (59.35%)	129 (63.86%)	
Employed	116 (65.17%)	154 (71.96%)		87 (40.65%)	73 (36.14%)	
Family annual per capita income (abbr. “FI”)			0.02			<0.01
Low (RMB < 10000)	59 (33.15%)	45 (21.03%)		99 (46.26%)	36 (17.82%)	
Middle (10000 ≤ RMB < 20000)	72 (40.45%)	92 (42.99%)		71 (33.18%)	74 (36.63%)	
High (RMB ≥ 20000)	47 (26.40%)	77 (35.98%)		44 (20.56%)	92 (45.54%)	
Personal position in family			<0.01			<0.01
High	83 (46.63%)	123 (57.48%)		79 (36.92%)	126 (62.38%)	
General	75 (42.13%)	87 (40.65%)		99 (46.26%)	73 (36.14%)	
Low	20 (11.24%)	4 (1.87%)		36 (16.82%)	3 (1.49%)	
Personal annual income (abbr. “PI”)			0.11			<0.01
No income (RMB = 0)	70 (39.33%)	83 (38.79%)		51 (23.83%)	49 (24.26%)	
0 < RMB < 10000	82 (46.07%)	83 (38.79%)		114 (53.27%)	69 (34.16%)	
RMB ≥ 10000	26 (14.61%)	48 (22.43%)		49 (22.90%)	84 (41.58%)	
Relative income in the family (PI vs. FI)			<0.01			0.30
PI < FI	169 (94.94%)	199 (92.99%)		174 (81.31%)	172 (85.15%)	
PI ≥ FI	9 (5.06%)	15 (7.01%)		40 (18.69%)	30 (14.85%)	
Mental disorders			<0.01			<0.01
Yes	70 (39.33%)	8 (3.74%)		117 (54.67%)	8 (3.96%)	
No	108 (60.67%)	206 (96.26%)		97 (45.33%)	201 (96.04%)	
Social support			<0.01			<0.01
Low	99(55.62%)	27 (12.62%)		145 (67.76%)	24 (11.88%)	
General	60 (33.71%)	92 (42.99%)		57 (26.64%)	87 (43.07%)	
High	19 (10.67%)	95 (44.39%)		12 (5.61%)	91 (45.05%)	
Coping strain			<0.01			<0.01
Low	15 (8.43%)	113 (52.80%)		23 (10.75%)	123 (60.89%)	
General	57 (32.02%)	87 (40.65%)		52 (24.30%)	69 (34.16%)	
High	106 (59.55%)	14 (6.54%)		139 (64.95%)	10 (4.95%)	

Number (*n*) and percentage for discrete variables. ^a^Mean and standard deviation (S.D.) for age.

**Table 2:** Suicide risk estimate for personal position in family and demographic factors.

Variables	Female		Male	
	OR	OR	OR	OR
	(1)	(2)	(3)	(4)
Personal position in family				
Low		7.09⁣^∗∗^ (2.22–22.62)		9.14⁣^∗∗^ (3.08–27.13)
General		1.08 (0.68–1.72)		1.69⁣^∗^ (1.02–2.80)
High		1		1
Age (years)				
≥25	1.84 (0.99–3.43)	1.77 (0.95–3.30)	3.10⁣^∗∗^ (1.63–5.89)	3.23⁣^∗∗^ (1.61–6.49)
<25	1	1	1	1
Educational level				
Primary school and below	5.46⁣^∗∗^ (2.78–10.71)	5.66⁣^∗∗^ (2.79–11.46)	10.26⁣^∗∗^ (5.04–20.86)	8.99⁣^∗∗^ (4.27–18.93)
Middle school	1.92⁣^∗^ (1.04–3.53)	1.96⁣^∗^ (1.03–3.73)	2.05⁣^∗^ (1.13–3.75)	2.10⁣^∗^ (1.12–3.93)
High school and above	1	1	1	1
Marital status				
Not currently married	2.33⁣^∗∗^ (1.22–4.42)	2.13⁣^∗^ (1.11–4.10)	4.65⁣^∗∗^ (2.48–8.71)	3.95⁣^∗∗^ (2.02–7.70)
Currently married	1	1	1	1
Working status				
Employed	1.56 (0.95–2.54)	1.68⁣^∗^ (1.02–2.76)	2.27⁣^∗∗^ (1.38–3.74)	2.20⁣^∗∗^ (1.32–3.68)
Unemployed	1	1	1	1
Family annual per capita income				
RMB < 10000	2.11⁣^∗^ (1.19–3.72)	1.98⁣^∗^ (1.12–3.51)	5.34⁣^∗∗^ (2.95–9.66)	4.50⁣^∗∗^ (2.45–8.28)
10000 ≤ RMB < 20000	1.24 (0.75–2.05)	1.13 (0.67–1.89)	2.10⁣^∗∗^ (1.21–3.66)	1.89⁣^∗^ (1.06–3.36)
RMB ≥ 20000	1	1	1	1
Observations	392	392	416	416

⁣^∗^*p* < 0.05; ⁣^∗∗^*p* < 0.01. OR means odds ratio; 95% CI in parentheses.

**Table 3:** The role of personal economic position in family on suicide risk.

Variables	Females				Males			
	OR	OR	OR	OR	OR	OR	OR	OR
	(1)	(2)	(3)	(4)	(5)	(6)	(7)	(8)
Personal position in family								
Low	7.09⁣^∗∗^ (2.22–22.62)	7.07⁣^∗∗^ (2.22–22.58)	7.12⁣^∗∗^ (2.24–22.65)	7.37⁣^∗∗^ (2.24–24.22)	9.14⁣^∗∗^ (3.08–27.13)	9.20⁣^∗∗^ (3.10–27.31)	9.15⁣^∗∗^ (3.08–27.19)	9.39⁣^∗∗^ (3.16–27.91)
General	1.08 (0.68–1.72)	1.08 (0.67–1.74)	1.09 (0.68–1.75)	1.08 (0.68–1.71)	1.69⁣^∗^ (1.02–2.80)	1.68⁣^∗^ (1.02–2.78)	1.69⁣^∗^ (1.02–2.79)	1.72⁣^∗^ (1.04–2.84)
High	1	1	1	1	1	1	1	1
Log (individual income)		1.00 (0.94–1.06)				0.99 (0.92–1.07)		
Have individual income or not								
Yes			1.02 (0.61–1.72)				0.99 (0.52–1.88)	
No			1				1	
Individual income higher than family average level or not								
Higher				1.88 (0.74–4.77)				0.80 (0.41–1.56)
Lower				1				1
Constant	0.12⁣^∗∗^ (0.05–0.29)	0.12⁣^∗∗^ (0.04–0.31)	0.11⁣^∗∗^ (0.04–0.30)	0.06⁣^∗∗^ (0.02–0.23)	0.02⁣^∗∗^ (0.01–0.07)	0.03⁣^∗∗^ (0.01–0.08)	0.02⁣^∗∗^ (0.01–0.07)	0.03⁣^∗∗^ (0.01–0.09)
Observations	392	392	392	392	416	416	416	416

⁣^∗^*p* < 0.05; ⁣^∗∗^*p* < 0.01. OR means odds ratio; 95% CI in parentheses. Other control variables included age, educational level, marital status, working status, and family annual income.

**Table 4:** Subgroup analysis by marital status and educational level.

	Personal position in family			*N*
	Low	General	High	
Panel A: by marital status				
Currently married	32.59⁣^∗∗^ (4.11–258.33)	1.67⁣^∗^ (1.09–2.58)	1	464
Not currently married	3.50⁣^∗^ (1.34–9.09)	0.94 (0.54–1.65)	1	344
Panel B: by educational level				
Primary school and below	17.33⁣^∗∗^ (2.66–112.82)	2.68⁣^∗∗^ (1.34–5.34)	1	233
Middle school	7.03⁣^∗∗^ (2.22–22.22)	1.07 (0.69–1.64)	1	436
High school and above	4.78 (0.92–24.89)	1.48 (0.53–4.16)	1	139

Note: We report OR and 95% CI in the table. Other control variables are same as those in Table 2. ⁣^∗^*p* < 0.05; ⁣^∗∗^*p* < 0.01.

**Table 5:** Analysis of potential mediators.

Variables	Female				Male			
	OR	OR	OR	OR	OR	OR	OR	OR
	(1)	(2)	(3)	(4)	(5)	(6)	(7)	(8)
Personal position in family								
Low	7.09⁣^∗∗^ (2.22–22.62)	3.81⁣^∗^ (1.06–13.70)	1.19 (0.31–4.55)	0.56 (0.11–2.91)	9.14⁣^∗∗^ (3.08–27.13)	9.60⁣^∗∗^ (2.94–31.27)	4.89⁣^∗^ (1.24–19.19)	3.71⁣^∗^ (1.01–13.68)
General	1.08 (0.68–1.72)	1.12 (0.68–1.84)	0.66 (0.38–1.16)	0.71 (0.38–1.32)	1.69⁣^∗^ (1.02–2.80)	1.72 (0.95–3.12)	1.12 (0.59–2.12)	1.06 (0.50–2.22)
High	1	1	1	1	1	1	1	1
Mental disorders								
Yes		13.36⁣^∗∗^ (5.86–30.44)	11.28⁣^∗∗^ (4.76–26.76)	11.70⁣^∗∗^ (4.29–31.94)		32.51⁣^∗∗^ (13.07–80.88)	21.00⁣^∗∗^ (8.37–52.70)	15.30⁣^∗∗^ (5.85–40.03)
No		1	1	1		1	1	1
Social support								
Low			16.50⁣^∗∗^ (7.46–36.45)	5.48⁣^∗∗^ (2.24–13.36)			22.34⁣^∗∗^ (8.71–57.32)	6.23⁣^∗∗^ (2.26–17.16)
General			2.71⁣^∗∗^ (1.36–5.40)	1.83 (0.85–3.90)			4.07⁣^∗∗^ (1.71–9.69)	2.94⁣^∗^ (1.18–7.29)
High			1	1			1	1
Coping strain								
Low				1				1
General				4.00⁣^∗∗^ (1.98–8.08)				2.37⁣^∗^ (1.08–5.17)
High				29.66⁣^∗∗^ (11.94–73.73)				18.19⁣^∗∗^ (6.10–54.24)
Other control variables	Yes	Yes	Yes	Yes	Yes	Yes	Yes	Yes
Observations	392	392	392	392	416	416	416	416

⁣^∗^*p* < 0.05; ⁣^∗∗^*p* < 0.01. OR means odds ratio; 95% CI in parentheses. Other control variables included age, educational level, marital status, working status, and family annual income.

**Table 6:** Decomposition using the KHB method.

	Female	Male
	OR	OR
Reduced	13.728⁣^∗∗^ (11.800)	34.052⁣^∗∗^ (23.181)
Full	0.563 (0.467)	4.118⁣^∗^ (2.809)
Diff	24.360⁣^∗∗∗^ (14.114)	8.268⁣^∗∗∗^ (3.894)

⁣^∗^*p* < 0.1; ⁣^∗∗^*p* < 0.05; ⁣^∗∗∗^*p* < 0.01. OR means odds ratio; robust standard error in parentheses.

**Table 7:** Disentangle contributions of mediators.

	Female		Male	
	Contribution to indirect effect (%)	Contribution to total effect (%)	Contribution to indirect effect (%)	Contribution to total effect (%)
Mental disorders (yes = 1)	29.94	36.50	32.62	19.53
Social support: low	17.28	21.07	16.30	9.76
Social support: general	7.83	9.54	4.45	2.67
Coping strain: low	52.79	64.35	45.34	27.15
Coping strain: general	-7.85	-9.57	1.29	0.77

## Data Availability

Data sharing is not applicable to this article as no new data were created or analyzed in this study.
